# Exploring the Knowledge, Attitudes, and Behavioural Responses of Healthcare Students towards Mental Illnesses—A Qualitative Study

**DOI:** 10.3390/ijerph17010025

**Published:** 2019-12-18

**Authors:** Taylor Riffel, Shu-Ping Chen

**Affiliations:** Department of Occupational Therapy, Faculty of Rehabilitation Medicine, University of Alberta, Edmonton, AB T6G 2G4, Canada; riffel@ualberta.ca

**Keywords:** mental illness stigma, healthcare students, mental health education

## Abstract

*Background:* The stigma of mental illness causes delays in seeking help, and often compromises victims’ therapeutic relationships with healthcare providers. The knowledge, attitudes, and behavioural responses of future healthcare professionals toward individuals with mental illnesses are explored here to suggest steps that will reduce mental illness stigma in healthcare providers. *Methods:* A generic qualitative approach—Qualitative Description—was used. Eighteen students from nine healthcare programs at a Canadian University participated in individual semi-structured interviews. Participants answered questions regarding their knowledge, attitudes, and behavioural responses towards individuals with mental illnesses. Thematic content analysis guided the data analysis. *Results:* Four main themes were constructed from the data: positive and negative general perceptions toward mental illness; contact experiences with mental illnesses; mental illness in a healthcare setting; and learning about mental illness in healthcare academia. *Conclusions:* Students showed well-rounded mental health knowledge and mostly positive behaviours toward individuals with mental illnesses. However, some students hold stigmatizing attitudes and do not feel prepared through their academic experiences to work with individuals with mental illnesses. Mental health education can reduce the stigma toward mental illness and improve the care delivered by healthcare professionals.

## 1. Introduction

The stigma ascribed to individuals with mental illnesses is not solely a concern among the general public. The incidence of mental health stigmatization in the healthcare system and among healthcare providers is surprising. Mental illness stigma can transpire at intrapersonal, interpersonal, and structural levels [1]. Individuals with mental illnesses frequently report feeling “devalued, dismissed, and dehumanized” [1] (p. 111) when coming into contact with health professionals.

Stigma is a multi-faceted construct and contributes to all aspects of a person’s life. According to the Mental Health Commission of Canada, “stigma is typically a social process, experienced or anticipated, characterized by exclusion, rejection, blame or devaluation that results from experience or reasonable anticipation of an adverse social judgment about a person or group” [2]. Such definition stresses that stigma is a negative social product and involves knowledge, emotional, and behavioral aspects. Stigma can be viewed from different perspectives in the literature of sociology, psychology, and medicine. Goffman (1963) first proposed three different types of stigma: (1) abominations of the body, related to physical deformities; (2) blemishes of individual character, such as weak will, mental disorder, or unemployment; and (3) tribal stigma of race, nation, or religion [3] (p. 4). The former two are also referred to as health-related stigma. Health-related stigma has both disease-specific and culture-specific characteristics [4]. Manifestations of negative perceptions, attitudes, and behaviors vary as they apply to different health problems in different environments. Among many health conditions, mental illness need to be addressed as a matter of priority because the general public seems to disapprove of persons with psychiatric disabilities more than persons with physical disabilities [5,6].

Corrigan and Watson, in 2002, proposed a social-cognitive model of public stigma. The model involves three components—stereotypes, prejudice, and discrimination—and demonstrates a stigma process. At the beginning, stereotypes are formed as an efficient means of categorizing information about social groups and represent a collective agreement regarding groups of persons. Stereotypes related to mental illness include dangerousness, character weakness, and incompetence. Persons who endorse these negative stereotypes would be prejudiced and generate negative emotional responses like anger or fear. Finally, stereotypes (the cognitive response) and prejudice (the emotional response) lead to discrimination (the behavioral magnification) [7].

The negative attitudes and viewpoints that many health professionals hold toward individuals with mental illnesses shape the quality of care that clients receive, and, ultimately, the quality of life of clients [8]. Stigmatizing behaviours and attitudes generate barriers such as “delays in help-seeking, discontinuation of treatment, suboptimal therapeutic relationships, patient safety concerns, and poorer quality mental and physical care” [1] (p. 112).

In 2006, the first Canadian national mental health report was released by the Standing Committee on Social Affairs, Science and Technology. In *Out of the Shadows at Last: Transforming Mental Health, Mental Illness, and Addiction Services in Canada*, the committee revealed a concern that healthcare professionals play a role in the perpetuation of mental illness stigma and can sabotage the process of recovery from mental illness by making negative assumptions and discriminating against those who have already been stigmatized by society [9]. There is a growing concern among mental health stigma researchers that healthcare professionals lack the educational background and awareness to work appropriately with individuals with mental illnesses [10].

While the problem of mental health stigma in healthcare professionals is apparent in the literature, the attitudes to and perceptions of mental illnesses by students in healthcare education programs are less apparent. Findings from Ogunsemi and colleagues [11] support these concerns. Responses were collected from final year medical students: one group answered a questionnaire that included a vignette with a psychiatric label attached, and a second group answered a questionnaire that included the same vignette with no psychiatric label. The study indicated that students showed negative attitudes toward individuals identified with a mental illness, and sought to maintain a significant social distance from individuals with this label [11]. A study conducted by Mukherjee and colleagues (2002) found that doctors and medical students shared similar negative attitudes toward individuals with mental illnesses, specifically individuals with schizophrenia, drug addiction, and alcohol addiction. More than 50% of the doctors and medical students surveyed felt that people with schizophrenia and drug and alcohol addictions were dangerous and unpredictable [12]. Another study revealed that healthcare providers stigmatized pregnant depressed women; particularly, some healthcare professionals were nervous around women with antenatal depression [13].

In contrast, not all research found negative attitudes and/or behaviours in healthcare professionals and students toward individuals with mental illnesses. For instance, mental health professionals have been found to hold a more positive attitude than the general public toward people with mental illnesses [8]. However, it was determined that mental health professionals tend to stereotype individuals with mental illnesses, especially individuals with schizophrenia, whom they consider to be particularly dangerous [8]. Henderson, et al., (2014) found that contact with people with mental illnesses was positively associated with more supportive attitudes in practitioners toward the civil rights of individuals with mental illnesses [14]. Another study demonstrated that primarily positive attitudes were held by entry-level physiotherapy undergraduate students toward psychiatry and individuals with mental illness [15].

Quantitative scales and assessments currently dominate the literature regarding the knowledge, attitudes, and behaviours of healthcare professionals and students toward mental illness. There is less focus on healthcare students’ perspectives than on healthcare professionals’ perspectives, and research articles considering this topic primarily focus on the nursing discipline, with less emphasis on other healthcare professions. The present study concentrates on the knowledge, attitudes, and behavioural responses of various healthcare students toward individuals with mental illnesses, since students represent the future quality of care received by such individuals. A qualitative inquiry will allow us to gather information while exploring context, recurring themes, and unforeseen findings.

## 2. Materials and Methods

### 2.1. Research Design

Qualitative research systematically collects and interprets material derived from conversations and observations. It is used to explore meanings of social phenomena as experienced by individuals in certain contexts [16]. This study used a generic qualitative approach—Qualitative Description [17]—which focuses on exploring how people interpret their experiences and the meaning they attribute to their experiences. The research methods within Qualitative Description are inductive and produce a low-inference description of a phenomenon in order to remain closer to the original data. Thematic analysis [18] was used to analyze and identify patterns and meaning and themes in the knowledge, attitudes, and behavioural responses of healthcare students in the context of mental illness. This research design is flexible, summarizes key qualities in a descriptive dataset, and provides insights into the data.

### 2.2. Participant Recruitment

Two students from each of nine healthcare programs at a Canadian University were recruited to participate in the interviews, for a total of eighteen students. These programs included: Dental Hygiene/Surgery, Dietetics/Nutrition, Medicine, Nursing, Occupational Therapy, Pharmacy, Physical Therapy, Psychology, and Speech-Language Pathology. Health science programs not considered to be in direct contact with clients with mental illnesses were not included in the study. Recruitment posters were released in approved and selected areas. All students interested in participating in the study contacted the researcher via email. Participants were chosen based on establishing the most variability (e.g., gender, year of study) in the sample population.

### 2.3. Data Collection

Participants in the study were interviewed to understand the context behind the knowledge, attitudes, and behavioural responses that healthcare students have toward mental illnesses. The interview procedure was semi-structured, involving a set of predetermined questions.

The study was approved by a University Research Ethics Board (Study ID: Pro00084418). Two students from each discipline were interviewed by the researcher separately. Each participant filled out a consent form and read information about the research study before the interview began. The interview lasted for a maximum of 60 min and followed the planned interview questions and follow up questions. Recruitment was planned to continue if the data from the initial eighteen interviews did not reach code saturation and meaning saturation.

During the interview, participants were asked a variety of questions about their experiences and opinions with respect to various elements of the project. They were asked questions regarding societal perceptions of mental illness and whether or not they believe mental illness stigma to be a concern in the community. The interview protocol included seven groups of questions: (1) What type of contact and experiences, in your personal life, have you had with individuals who have mental health issues? (2) What is your understanding of mental illnesses? (3) How would you describe your degree of compassion and empathy towards individuals with mental illnesses? (4) What would you do if you had a mental illness? (5) Since starting your healthcare program, what type of experience(s) have you had working with individuals with mental illnesses? (6) Since starting your healthcare program, what type of information have you received in your academic learning regarding mental illness? And (7) Based on your overall personal and academic experiences and knowledge of mental illnesses, how would you feel working with individuals who have mental illness? Participants were informed that there were no right or wrong answers. All interviews were audio recorded for data analyses.

### 2.4. Data Analysis

All interview data were transcribed verbatim. The analyses involved structured coding procedures and thematic characterizations of the coded segments [19]. After reading through the text, the lead researcher (TR) and the research supervisor (SC) analyzed the transcriptions using the coding reliability approach of thematic analysis [18]. Initial coding was performed by TR and SC independently, using both inductive and deductive approaches [20]. Relevant codes were identified, and a structured codebook was developed following the procedures outlined by MacQueen et al. [21]. The codebook included detailed definitions, typical exemplars, atypical exemplars, and marginal/irrelevant examples from the texts to illustrate the range of meanings assigned to themes. After the initial coding was complete, the core analysis included code categorization and thematic comparison [19]. Both researchers determined the final coding through consensus, reviewed the patterns of shared meanings, and categorized overlapping themes into a practicable list of defined themes.

## 3. Results

### 3.1. Participants

A total of 18 students from nine healthcare programs at a Canadian University were interviewed. The demographics of participants are summarized in Table 1. The majority (67%) of participants were female, and most participants were in their first year (22%) and second year (50%) of study in their current programs. Approximately 27% of participants had completed a previous postsecondary program in addition to their current healthcare program. Of the 18 participants, nine individuals reported mental health concerns themselves (diagnosed or undiagnosed).

**Figure 1:**
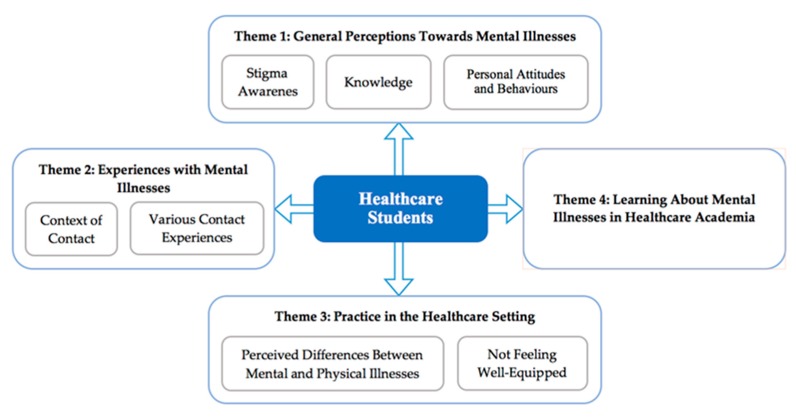
Outline of Themes and subthemes.

Four main themes were constructed from the data: general perceptions of mental illnesses, contact experiences with mental illnesses, mental illnesses in a healthcare setting, and mental health education in healthcare academia (see Figure 1).

### 3.2. General Perceptions of Healthcare Students toward Mental Illnesses

Participants were asked questions regarding their knowledge and general perceptions of mental illnesses and mental illness stigma in society. Participants discussed what they believed to be the origin of mental illnesses and the best treatments for treating mental disorders.

#### 3.2.1. Stigma Awareness

Most participants were cognizant of the stigma toward mental illness in society. They identified society as having either positive or negative perceptions toward mental illnesses. Positive perceptions included the belief that in more recent generations there is more understanding and acceptance of mental illnesses. Additionally, some participants noted that mental illness stigma is being broken down, for example, “I believe there is a stigma, but it’s slowly going away, fading away, I would hope. I don’t think there is much of a stigma anymore” (Participant 12).

Other participants identified harmful perceptions and responses toward mental illnesses in society. One participant noted that, although there has been increased awareness of mental illnesses, there has also been a noted lack of action to combat stigma. Participant 16 stated that mental illness is becoming “glorified” in the community, for example, people like to share that “oh I write this sad poetry,” “I’m so broken,” or “somebody help me.” “People will go on social media and try and post their problems on there, which there’s nothing wrong with that, but it’s just like garnering like pity almost” (Participant 16). Numerous participants identified society as having negative perceptions toward mental illnesses. They noted that many people viewed individuals with mental illnesses as being “unstable,” “unpredictable,” “confused,” and “scary”.

Both positives and negatives were cited by participants in regard to the media’s impact on mental illness stigma. Two participants thought that the media has a positive influence on the diminution of mental illness stigma. Participant 2 stated, “… there is better awareness through these campaigns, there is more understanding and people can be more vocal about it through Facebook, through the social media avenues.” Conversely, several participants discussed the harmful impact that the media has on mental illness stigma. Participant 17 noted, “… you see people in horror films, and things like that, who are having those more unspoken mental disorders and they are doing awful things which is not the case usually, but it’s what people see”.

#### 3.2.2. Knowledge of Mental Illnesses

Participants appeared to have a rudimentary understanding of mental illnesses. The majority of participants were able to identify some causes of mental illnesses, such as genetics, social/environmental conditions, and biological/medical disorders. Many participants thought that trauma, stress, and lack of resiliency could cause mental illnesses. Participants were able to identify several treatments for mental illnesses, including medication, therapy, social support, and lifestyle changes. Almost half of the participants noted that treatment is person-dependent and that there is no one-size-fits-all approach to mental illness treatment.

#### 3.2.3. Personal Attitudes and Behaviours toward Mental Illnesses

Participants had mixed perceptions of mental illness diagnoses or “labels.” Some participants believed that it was a positive to have a label as it helps the individual to find appropriate resources and support. Others thought that a label can be seen as a weakness and might also bias people against the troubled individual. One participant thought: “if you’re labelled with a mental illness, people look down on you as weakness …” (Participant 2).

When considering whether the participant would feel comfortable disclosing a hypothetical mental illness to others, all participants stated that they would feel comfortable disclosing to family members, friends, a significant other, and/or a professional. However, the majority of participants stated they would not disclose a hypothetical mental illness in a work setting (such as a job interview) and/or a school setting: “I would say I’m a private person, so I probably would not feel comfortable [disclosing] outside a close circle of family and friends, so not at school or at work” (Participant 4).

Participants opinions varied with regard to their comfort around individuals with mental illnesses. Many stated that they would be comfortable around less severe and more familiar mental illnesses, such as anxiety and depression. “… I think I’m a lot more familiar with [anxiety and depression], because that’s something that, like, I’ve seen people go through more often” (Participant 5). Others shared that they would be fearful of their safety or would be uncomfortable around someone with a mental illness. “… honestly, kind of a little scared, because like I don’t know what they’re going to do” (Participant 16).

All participants stated that they would be supportive toward an individual who was being bullied for a mental illness. Two participants indicated that their support would depend on their closeness with the bullied individual. All participants agreed that they would be understanding and/or supportive if their significant other disclosed a mental illness to them.

### 3.3. Healthcare Students’ Experiences with Mental Illnesses

Participants’ contacts with mental illnesses included self-experience with mental illnesses, having a loved-one with a mental illness, or knowing individuals (at school, work, or during volunteer experiences) that have experienced mental illness. Participants’ experiences with mental illnesses depended on the context of the contact.

#### 3.3.1. Context of Contact

Some participants had known classmates with mental illnesses, and some had interacted with mentally ill individuals in work or volunteer situations. All but one of the participants reported having either a self-experience with mental illnesses, or experiencing a family member, a friend, or a significant other with a mental illness. One participant expressed: “My own personal experience in the mental health system having struggled with mental health so I have met individuals that way and in just having friends and family with also various diagnoses” (Participant 9).

#### 3.3.2. Contact Experience

The participants’ contact experiences led to positive transformations and some difficulties. Most participants reported positive transformations through contact with individuals with mental illnesses, such as changes in worldview/perception and the development of empathy. The difficulties included being unable to determine when a mental illness is in fact a mental illness, and the negative impact of mental illness on family members (e.g., parents’ worrying, children being impressionable). One participant explained that it is difficult to know when someone truly suffers from a mental illness: “But it’s drawing that line personally where I’m convinced that they’re having an issue versus just like making it an issue, rather than it actual(ly) being something” (Participant 13).

### 3.4. Practice in the Healthcare Setting

Participants discussed barriers to the treatment of mental illness compared to the treatment of physical illnesses in the healthcare system.

#### 3.4.1. Perceived Differences between Physical and Mental Illnesses

Participants’ perceptions of treating mental illness were more negative than their perceptions of treating physical illness. Some participants attributed this to the invisibility and unpredictability of mental illness. One participant stated: “But for mental illness, I think it requires more effort … because it’s not visible to the eye. … you don’t know if a mental patient is ever going to snap again” (Participant 3). Mental illness was seen by participants as more permanent, more long-lasting, rooted in curses and religion, and facing more stigma in society than physical illness, all of which made it more difficult to treat than physical illness.

#### 3.4.2. Not Feeling Well-Equipped

Participants were familiar with common mental illnesses such as anxiety and depression, but many reported having less experience handling patients with severe mental illnesses. Therefore, they did not feel prepared to provide support for such individuals. One participant stated: “… I’ve got the tools to initially identify things and think okay, maybe this is something that needs to be addressed, but I need a lot more education before I’d be able to genuinely help someone” (Participant 15).

### 3.5. Learning about Mental Illnesses in Healthcare Academia

Most participants considered the mental illness information taught in their healthcare programs to be insufficient. Participant 10 described the deficit in his program: “I know that two years is a short period of time, so we can’t be like fully competent at the end of it. But at the same time I definitely feel that there’s a huge deficit.” Participants’ suggestions to improve their programs included more emphasis on mental health, teachings about mental illness provided earlier in the program, more student experiences with clients that have mental illnesses, and an effort by instructors to destigmatize and demystify mental illness. One participant commented: “I think really the experience is the main thing … having students work with clients with mental health [problems] from the start of their program, might be helpful” (Participant 6).

## 4. Discussion

This study of healthcare students’ experiences with individuals with mental illnesses increases our understanding of current healthcare programs in the sector. Healthcare student participants had a basic understanding of mental illnesses and recognized an assortment of mental illness origins and treatments. Participants generally held positive attitudes toward individuals with mental illnesses, however, several stigmatizing perceptions were evident in the study findings.

Participants identified positive and stigmatizing perceptions of mental illnesses currently held by society, although some participants acknowledged that mental illness stigmatization has lessened in recent years. No mention or acknowledgement of any personal stigma was made by the students in the study. Although most of the participants did not share society’s stigmatizing views of mental illness, some of the participants presented fearful, uncomfortable, and stigmatizing attitudes toward mental illnesses. The finding that some healthcare students hold stigmatizing attitudes toward individuals with mental illnesses is not new. The healthcare literature documents stigmatizing attitudes and behaviours across the healthcare spectrum [1]. Our findings are novel with respect to the absence in some healthcare students of acknowledged personal stigmatizing attitudes toward individuals with mental illnesses.

Interestingly, social media influences human interaction in the era of technology. In this study, the phenomenon that people use social media to post their mental health problems was somehow interpreted as an attempt to garner pity or attention. This interpretation may potentially be a type of stigma. The interaction between users of social media and their emotional experience is complex [22]. As frequently posting positive or negative effects on social media has been associated with depression [23], it is important to further understand the theoretical mechanism of social media posts—the difference between a call for help and a plea for attention—and its association with stigmatized attitudes.

Half of the participants in this study had experienced a mental illness themselves. This proportion is considerably higher than the Canadian national average of 20%, thus there is a question of whether there is a higher amount of mental illness among healthcare students or whether participants who have experienced a mental illness themselves or in others were simply more motivated to enroll in this study [24]. Other studies have shown a high prevalence of mental illness among workers in the healthcare industry [25]. However, literature considering the levels of mental illness apparent in healthcare students was not found at this time. The presence of mental illness among healthcare students and professionals is important, because healthcare providers are responsible for providing competent and safe care to the public [26]. Interestingly, participants who had lived experiences of mental illness held less of an “us and them” discrepancy between themselves and people with mental health disorders. Thus, having self-experience with mental illness might decrease mental illness stigmatization and foster empathy and understanding between healthcare workers and mentally ill individuals.

All participants in this study, except for one, had experienced direct contact with mental illnesses outside their healthcare practice. The majority of these participants mentioned being positively impacted through such contact, and thus had an increased awareness of the problems mental illnesses entail. This is consistent with findings that direct contact experiences with individuals with mental illnesses can help to reduce stigma [27]. Of note, some participants discussed challenges they faced when a loved one experienced mental illness, such as stress on the caregiver and a negative impact on family relations. This is consistent with results in the literature that noted similar stressors and emotional concerns among individuals providing support and care to a loved one with a mental illness [28]. On the other hand, healthcare students who, in their private life, have experienced difficulties with individuals with mental illnesses may have a negative outlook toward mental health, that affects their behaviour in their healthcare practice.

When discussing the differences between treating mental versus physical illness, most participants stressed the complexity of treating mental illness and the stigmatization that mental illness faces in society. The findings regarding participants’ stigma toward mental illness versus their nonstigmatic approaches to physical illness are consistent with the findings in other studies [29,30]. For instance, Corrigan and colleagues [29] determined that mental disorders are often more stigmatized than physical disorders. The present study suggested that these perceptions stemmed from the fact that mental illness is often more difficult to treat, more invisible, and more long-lasting than physical illness. Such perceptions might lead healthcare students to avoid working in a mental healthcare setting in future.

Most participants reported that their healthcare program did not provide sufficient mental illness education to adequately prepare them to provide appropriate support for individuals with mental illnesses. This echoes Tognazzini and colleagues’ [10] finding that healthcare professionals may be deficient in mental illness education and awareness. Many participants commented that, although they did not currently feel adequately prepared, they hoped to receive more mental health education and experience during their program. Healthcare programs can help to improve students’ mental health education by delivering mental health training earlier on in the program and throughout it, providing more integration with clients with mental illnesses, clarifying the scope of the discipline when working with individuals with mental illnesses, and demystifying/destigmatizing mental illness as an “impossible” illness to work with. According to the Mental Health Commission of Canada [26], healthcare workers are 1.5 times more likely than workers in other sectors to require time off work due to an illness or disability. Therefore, healthcare students should be taught not only how to work with various mental illnesses, but also how to develop appropriate coping strategies for their own mental health before entering the workforce. The health and well-being of healthcare professionals can assist in helping to reduce stigma in the healthcare system and would have a positive impact on staff and patient safety [1].

### 4.1. Strengths and Limitations

The small-scale qualitative design (i.e., a total of 18 participants in a single university) limits the generalizability of the findings in terms of their being representative of healthcare student populations and healthcare educational programs. However, the diversity of healthcare student participants recruited was a strength of the study. Participant diversity was based on program, gender, and year of study. Participant biases included acquiescence bias and social desirability bias. To minimize the effects of these biases on the results, questions were open-ended, and participants were informed that there was no right or wrong answer to any question and were told that they could advise the interviewer if they did not want to answer a question.

The main causes of researcher bias would be leading questions and wording biases. The learning curve of the researcher in training (TR) must be considered in the conduction of interviews. TR was trained by an experienced researcher (SC) to avoid using leading questions and to transcribe participants’ answers verbatim; however, mastery of these skills requires practice and experience.

### 4.2. Implications for Future Research

This study had a small sample size (two participants) taken from each of the nine faculties. Therefore, differences between the healthcare programs were not analyzed in this study. Future research could use variations between disciplines to inform specific mental health education in healthcare programs. An in-depth analysis of the mental health education taught and the experiences (or lack thereof) encountered in each faculty would help to determine whether there is a correlation between healthcare program teachings and the knowledge, attitudes, and behavioural responses of healthcare students.

Future research could find interventions and strategies that reduce the stigma related to mental illness among students in healthcare programs. For example, contact-based education, where healthcare students engage with individuals with mental illness(es) [31], would acclimatize students and reduce the mystery of the diseases.

## 5. Conclusions

Mental illness stigma is evident in healthcare settings. Quantitative research tends to dominate this area of literature, with less focus on qualitative understanding. Based on the findings in this research and previous studies, some students in healthcare programs hold stigmatizing perceptions of individuals with mental illnesses. This research indicates that more work needs to be done to familiarize healthcare students with mental health issues and to accustom healthcare students to working with mentally ill individuals.

## Figures and Tables

**Table 1:** Demographic characteristics of study participants.

Participant Identifier	Gender	Healthcare Program	Year of Study
Participant 1	Female	Dentistry	Second
Participant 2	Female	Dentistry	Second
Participant 3	Female	Dietetics/Nutrition	First
Participant 4	Female	Dietetics/Nutrition	Third
Participant 5	Male	Medicine	First
Participant 6	Female	Medicine	First
Participant 7	Female	Nursing	Second
Participant 8	Male	Nursing	Third
Participant 9	Female	Occupational Therapy	Second
Participant 10	Male	Occupational Therapy	Second
Participant 11	Female	Pharmacy	First
Participant 12	Male	Pharmacy	Fourth
Participant 13	Male	Physiotherapy	Second
Participant 14	Male	Physiotherapy	Second
Participant 15	Female	Psychology	Fifth
Participant 16	Female	Psychology	Second
Participant 17	Female	Speech-Language Pathology	First
Participant 18	Female	Speech-Language Pathology	Second

## References

[1] Knaak S., Mantler E., Szeto A. (2017). Mental illness-related stigma in healthcare: Barriers to access and care and evidence-based solutions. Healthc. Manag. Forum.

[2] Martin N., Johnston V. (2007). A Time for Action: Tackling Stigma and Discrimination.

[3] Goffman E. (1963). Stigma: Notes on the Management of Spoiled Identity.

[4] Weiss M.G., Ramakrishna J. (2004). Health-related stigma: Rethinking concepts and intervention. Research Workshop on Health-Related Stigma and Discrimination.

[5] Piner K., Kahle L. (1984). Adapting to the stigmatizing label of mental illness: Forgone but not forgotten. J. Personal. Soc. Psychol..

[6] Weiner B., Perry R.P., Magnusson J. (1988). An attributional analysis of reactions to stigmas. J. Personal. Soc. Psychol..

[7] Corrigan P.W., Watson A.C. (2002). The paradox of self-stigma and mental illness. Clin. Psychol. Sci. Pract..

[8] Stuber J.P., Rocha A., Christian A., Link B.G. (2014). Conceptions of mental illness: Attitudes of mental health professionals and the general public. Psychiatr. Serv..

[9] The Standing Committee on Social Affairs, Science and Technology Canada (2006). Out of Shadows at Last: Transforming Mental Health, Mental Illness, and Addiction Services in Canada. https://www.mentalhealthcommission.ca/sites/default/files/out_of_the_shadows_at_last_-_full_0_0.pdf.

[10] Tognazzini P., Davis C., Kean A., Osborne M., Wong K.K. (2008). Reducing the stigma of mental illness. Can. Nurse.

[11] Ogunsemi O.O., Odusan O., Olatawura M.O. (2008). Stigmatising attitude of medical students toward a psychiatry label. Ann. Gen. Psychiatry.

[12] Mukherjee R., Fialho A., Wijetunge A., Checinski K., Surgenor T. (2002). The stigmatization of psychiatric illness: The attitudes of medical students and doctors in a London teaching hospital. Psychiatr. Bull..

[13] Gawley L., Einarson A., Bowen A. (2011). Stigma and attitudes toward antenatal depression and antidepressant use during pregnancy in healthcare students. Adv. Health Sci. Educ..

[14] Henderson C., Noblett J., Parke H., Clement S., Caffrey A., Gale-Grant O., Schulze B., Druss B., Thornicroft G. (2014). Mental health-related stigma in health care and mental health-care settings. Lancet Psychiatry.

[15] Connaughton J., Gibson W. (2016). Physiotherapy students’ attitudes toward psychiatry and mental health: A cross-sectional study. Physiother. Can..

[16] Grossoehme D.H. (2014). Research metholodology overview of qualitative research. J Health Care Chaplain.

[17] Kahlke R.M. (2014). Generic Qualitative Approaches: Pitfalls and benefits of methodological mixology. Int. J. Qual. Methods.

[18] Braun V., Clarke V. (2006). Using thematic analysis in psychology. Qual. Res. Psychol..

[19] Benard H.R., Wutich A.Y., Ryan G.W. (2016). Analyzing Qualitative Data: Systematic Approaches.

[20] Greg G., Macqueen K.M., Namey E.E. (2012). Applied Thematic Analysis.

[21] MacQueen K.M., McLellan E., Kay K., Milstein B. (1998). Codebook development for team-based qualitative analysis. Cult. Anthropol. Methods.

[22] Seabrook E.M., Kern M.L., Rickard N.S. (2016). Social networking sites, depression, and anxiety: A systematic review. JMIR Ment. Health.

[23] Locatelli S.M., Kluwe K., Bryant F.B. (2012). Facebook use and the tendency to ruminate among college students: Testing mediational hypotheses. J. Educ. Comput. Res..

[24] Mental Health Commission of Canada (2013). Making the Case for Investing in Mental Health in Canada. https://cmha.ca/about-cmha/fast-facts-about-mental-illness.

[25] Kim M.S., Kim T., Lee D., Yook J.H., Hong Y.C., Lee S.Y., Yoon J.H., Kang M.Y. (2018). Mental disorders among workers in the healthcare industry: 2014 national health insurance data. Ann. Occup. Environ. Med..

[26] Mental Health Commission of Canada (2016). Issue Brief: Workplace Mental Health.

[27] Nguyen E., Chen T.F., O’Reilly C.L. (2012). Evaluating the impact of direct and indirect contact on the mental health stigma of pharmacy students. Soc. Psychiatry Psychiatr. Epidemiol..

[28] Shamsaei F., Cheraghi F., Esmaeilli R. (2015). The family challenge of caring for the chronically mentally ill: A phenomenological study. Iran. J. Psychiatry Behav. Sci..

[29] Corrigan P.W., River L.P., Lundin R.K., Penn D.L., Uphoff-Wasowski K., Campion J., Mathisen J., Gagnon C., Bergman M., Goldstein H. (2001). Three strategies for changing attributions about severe mental illness. Schizophr. Bull..

[30] Ahmedani B.K. (2011). Mental health stigma: Society, individuals, and the profession. J. Soc. Work Values Ethics.

[31] Pattern S.B., Remillard A., Philips L., Modgill G., Szeto AC H., Kassam A., Gardner D.M. (2012). Effectiveness of contact-based education for reducing mental illness-related stigma in pharmacy students. BMC Med. Educ..

